# Intelligence, Correspondence, &c.

**Published:** 1834-10-01

**Authors:** 


### INTELLIGENCE, CORRESPONDENCE, &c.

Reviews or notices of several works have been unavoidably postponed. Amongst others, the Provincial Transactions, and Mr. Simpson upon Education, which were promised for this number, must stand over for our next.

Two of the communications of Dr. Kennedy were too late. One, he will perceive, is inserted.

Dr. Forsayth was also too late for the present number. His case will appear in our next.

We are compelled to defer a notice of Mr. Stevenson's operation for cataract. We lately had the pleasure of seeing him perform it:—the dexterity was great, and we believe the result has been successful.

On the 1st of January, 1835, will be published *Professor Grant's Outlines of Comparative Anatomy, in one volume,* 8vo, with upwards of one hundred woodcuts from drawings on wood by Mr. H. J. Townsend, engraved by Branston.

